# Incidence trends in bladder and lung cancers between Denmark, Finland and Sweden may implicate oral tobacco (snuff/snus) as a possible risk factor

**DOI:** 10.1186/s12885-021-08371-w

**Published:** 2021-05-25

**Authors:** Kari Hemminki, Asta Försti, Akseli Hemminki, Börje Ljungberg, Otto Hemminki

**Affiliations:** 1 Biomedical Center, Faculty of Medicine and Biomedical Center in Pilsen, Charles University in Prague, 30605 Pilsen, Czech Republic; 2 Division of Cancer Epidemiology, German Cancer Research Center (DKFZ), Im Neuenheimer Feld 580, D-69120 Heidelberg, Germany; 3 Hopp Children’s Cancer Center (KiTZ), Heidelberg, Germany; 4 Division of Pediatric Neurooncology, German Cancer Research Center (DKFZ), German Cancer Consortium (DKTK), Heidelberg, Germany; 5 Cancer Gene Therapy Group, Translational Immunology Research Program, University of Helsinki, Helsinki, Finland; 6 Comprehensive Cancer Center, Helsinki University Hospital, Helsinki, Finland; 7 Department of Surgical and Perioperative Sciences, Urology and Andrology, Umeå University, Umeå, Sweden; 8 Division of Urologic Oncology, Department of Surgical Oncology, Princess Margaret Cancer Center, University Health Network and University of Toronto, Toronto, Ontario Canada; 9 Department of Urology, Helsinki University Hospital and University of Helsinki, Helsinki, Finland

**Keywords:** Risk factors, Sex difference, Incidence trend, Tobacco products, Snuffing

## Abstract

**Background:**

The dominant risk factor for urinary bladder cancer has been cigarette smoking, but, as smoking prevalence is decreasing in many populations, other risk factors may become uncovered. Such new risk factors could be responsible for halting the declining incidence of bladder cancer. We hypothesize that snuff use by Swedish men may increase the rate for bladder cancer, as snuff contains carcinogenic nitrosamines.

**Methods:**

We carried out an ecological study by comparing incidence trends in lung and bladder cancers between Danish, Finnish and Swedish men in order to test if the Swedish bladder cancer rate deviates from the Danish and Finnish ones. We used the NORDCAN database for cancer data from 1960 through 2016 to test the hypothesis.

**Results:**

In the three countries, the incidence of lung cancer started to decrease after a peak incidence, and this was later followed by declining incidence in bladder cancer in Denmark from 1990 to 2016 by 14.3%, in Finland by 8.3% but not in Sweden (the decline of 1.4% was not significant). The difference in trends can be partly explained by the increasing incidence in Swedish men aged 70 or more years. Sweden differs from the two other countries by low male smoking prevalence but increasing use of snuff recorded by various surveys.

**Conclusion:**

The stable bladder cancer trend for Swedish men was opposite to the declining trends in Denmark, Finland and globally. We suggest that this unusual finding may be related to the increasing use of snuff by Swedish men. Average users of snuff are exposed to at least 3 times higher levels of carcinogenic tobacco-specific nitrosamines than a smoker of one daily pack of cigarettes.

**Supplementary Information:**

The online version contains supplementary material available at 10.1186/s12885-021-08371-w.

## Introduction

The incidence trends of lung and urinary bladder cancers have marked the cigarette smoking epidemic which started in various countries before or after World War II [1, 2]. The incidence rates followed the consumption of cigarettes with a lag time of 20 to 40 years and have started to decline as the number of smokers has diminished [3, 4]. Relative risks for tobacco-related lung cancer are of the order of 10 to 20 in active smokers compared to non-smokers, depending on pack-years smoked and other factors, and they remain at levels of 3–5 after 20 years of quitting [5, 6]. Risk of bladder cancer is about 2–4 times higher for active smokers [5, 7–10]. In former smokers the risk is around 2 fold higher, depending on the time since quitting, and it may take at least 30 years to reach the incidence level of non-smokers [5, 7–9]. The changing composition of cigarettes and tobacco have apparently lowered the risk of lung cancer by about 20% but increased the risk of bladder cancer by 30% [11, 12]. Other risk factors for these cancers include occupational exposures, air pollution, type 2 diabetes and family history [10, 13–16]. As the proportion of smokers decreases, it is likely that cancer trends at smoking related sites will be influenced by weaker risk factors prevalent in the population.

A unique aspect of smoking habits among Swedish men is that their smoking prevalence decreased earlier than in other European countries, resulting in the lowest lung cancer incidence at least since 1980 [17, 18]. Lung cancer rates for Danish and Finnish men decreased slower but, in contrast to Swedish men, they did not take up the habit of oral tobacco (snuff, locally called ‘snus’) use. The use of snuff increased in Sweden antiparallel to smoking and in the early 1990s daily snuff users (20%) passed daily smokers in prevalence [19]. Several studies have examined possible carcinogenic effects of the Swedish snuff. Recent studies reported a small overall excess mortality in cancer and risk of rectal but not of colon cancer [20, 21]. Studies on oral and pancreatic cancers have been negative [22, 23]. US users of smokeless tobacco have an increased risk of oral and bladder cancers [24].

We carried out an ecological study by comparing the incidence change between lung and bladder cancers between Danish, Finnish and Swedish men in order to test if the Swedish bladder cancer trend deviates from the Danish and Finnish ones, hypothesizing that snuff use may increase the rate for bladder cancer. We used the NORDCAN database, originating from the local cancer registries, to test the hypothesis.

## Materials and methods

### Tobacco use by Danish, Finnish and Swedish men

The three Nordic countries (Denmark, Finland and Sweden) have quite different past patterns of smoking, including among the highest male smoking prevalence in Europe for Denmark and Finland, and the lowest prevalence in Sweden (www.pnlee.co.uk/ISS.htm) [25, 26]. World War II boosted smoking among Finnish men, as each solder was entitled to a daily ration of 5 cigarettes; the habit continued and smoking prevalence among Finnish men remained at 60% until 1960. Yet the prevalence was lower than for Danish men and somewhat higher than for Swedish men (www.pnlee.co.uk/ISS.htm) [27, 28]. Subsequently, smoking prevalence declined fastest for Swedish and slowest for Danish men [17]. Of particular interest in this comparison is the variable use of oral tobacco (snuff/snus) by men in these countries. While Swedish men reduced their smoking level from 28% (daily smokers) in 1988/89 to 15% in 2004/05, daily snuff users increased from 19 to 27% in the same period [19]. More than half of daily snuff users were non-smokers. The highest prevalence of snuff users (rather stable at 30%) was in men aged 20 to 29, and among older men snuff use increased steadily. Among Finnish men, 36% were daily smokers in 1988/89, which deceased to 28% in 2004/05 [19]. Daily consumption of snuff was recorded only for 2004/05 at 3%. Snuff use has also been low in Denmark.

### Data analysis

We used the NORDCAN database which is a compilation of data from the high-level Nordic cancer registries as described [29] (https://NORDCAN.iarc.fr/en/database#bloc2). In the database, bladder cancer is part of urothelial cancers covered by the codes C65–68 (cancers of the pelvis, ureter, bladder), D09.0–1, D30.1–9, D41.1–9 (in situ and tumors of undefined behavior at these sites). Coding practices for bladder cancer have been internationally variable as far as consideration of benign lesions. In NORDCAN these lesions were included retroactively in 2015; however it is unclear how uniform the classification of benign lesions was back in time [30]. Nevertheless, the historic incidence data for bladder cancer show no abrupt changes over time.

The vast majority of urothelial cancers are located in the bladder (90–95% of all) with the upper urinary tract (renal pelvis and ureter) accounting for the rest [31]. For simplicity and the dominance of a single entity, we call these cancers as ‘bladder cancer’. Urothelial cancers share risk factors, including smoking [5, 31, 32].

For incidence analysis, the world standard population was used in age adjustment. In incidence diagrams 3-year smoothing was used because of small case numbers; in smoothing, moving averages are calculated for each data point over 3 years. In assessing incidence trends estimated annual percentage change (EAPC) was used to describe the magnitude of change in the trend on fitting a regression model (shown in some figures as a dotted line) to the log of the age standardized incidence rate. This described the average annual rate change (%) over the time period selected.

## Results

**Fig. 1:**
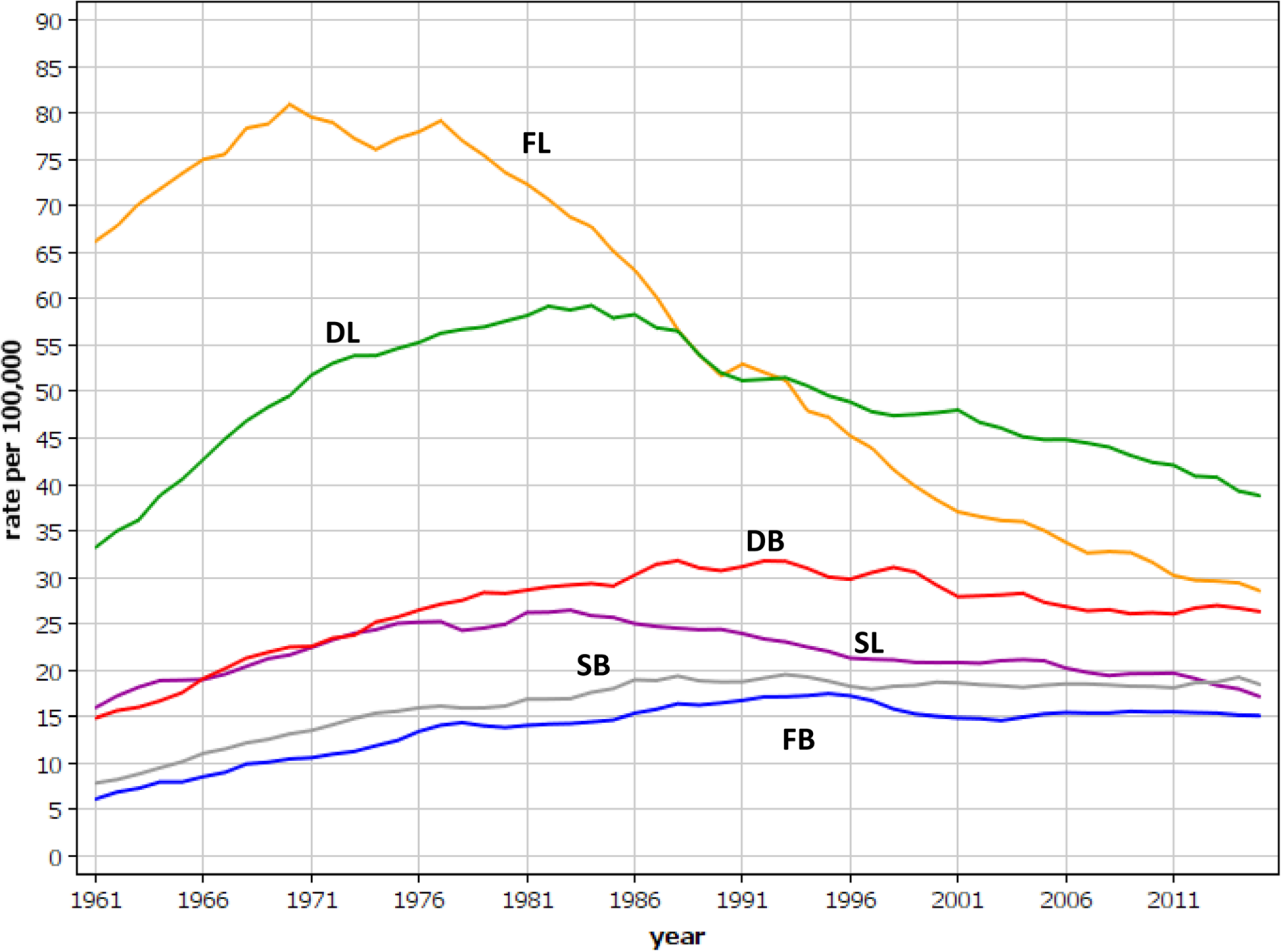
Age-standardized incidence rates for Danish, Finnish and Swedish male lung and bladder cancer between 1960 and 2016. Lung cancer curves for Danish, Finnish and Swedish men are shown by DL, FL and SL, respectively, and for bladder cancers accordingly DB, FB and SB

Age-standardized incidence rates for lung and bladder cancers in Nordic males are shown in Fig. 1. The highest incidence in lung cancer was recorded for Finland, reaching a maximum around 1970. The Danish and Swedish incidence peaked in the early 1980s. The rates for bladder cancer deviated from those of lung cancer. Increasing incidences were noted in the three countries until the 1990s; the Finnish rates remained the lowest and the Danish rates the highest throughout the observation period. While the incidence of lung cancer was much higher than that of bladder cancer, the rate differences declined over the course of time, being 2 fold in Finland and 1.5 fold in Denmark in the 2010s, when the Swedish bladder cancer incidence surpassed that of lung cancer.

**Fig. 2:**
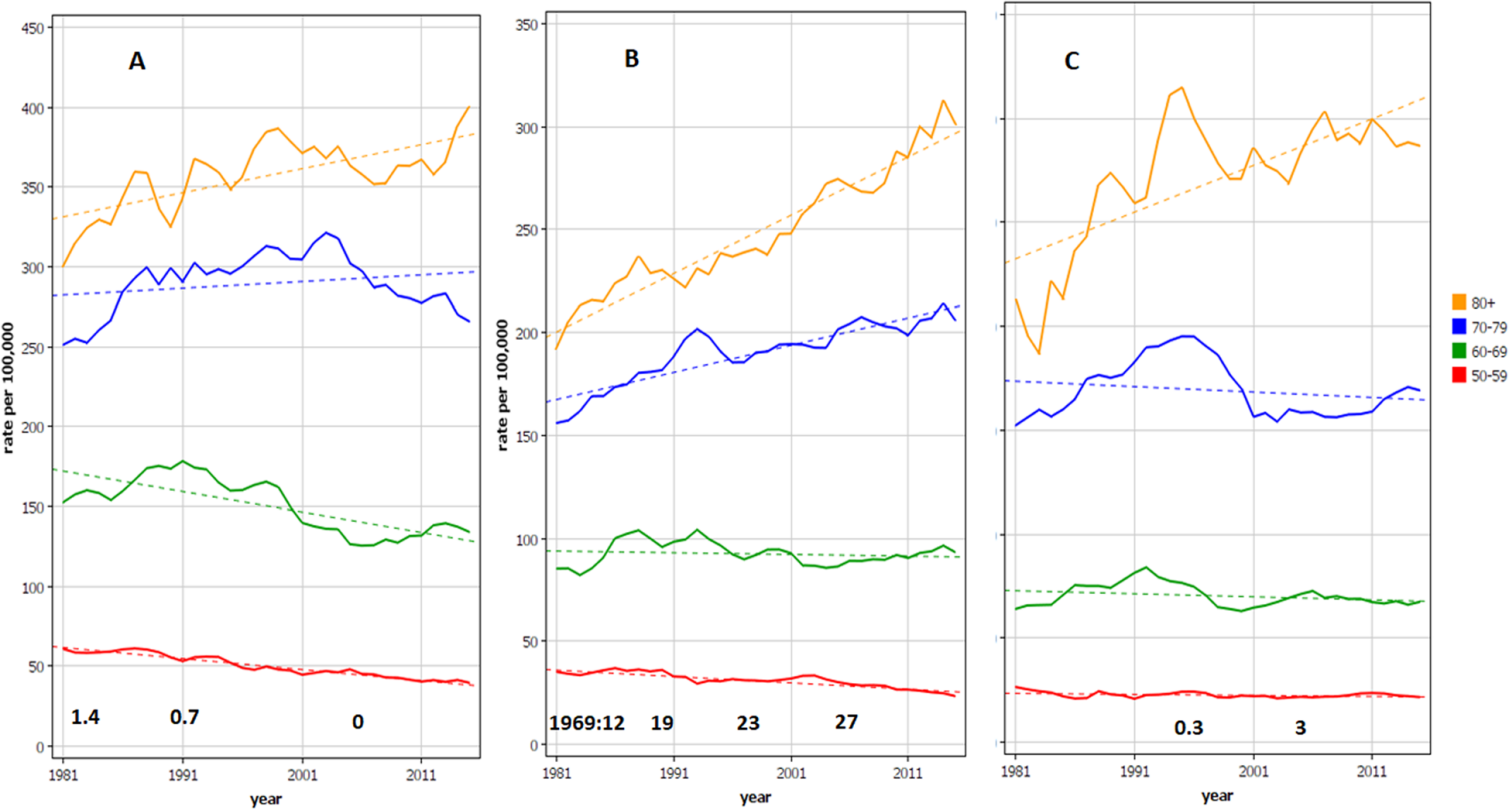
Age-specific incidence rates for bladder cancer in Danish **A**, Swedish **B** and Finnish **C** men from 1980 through 2016. The dotted line shows a regression line fitted to the log of the rate. Note that the y-axis scale for Denmark extend to 450/100,000 compared to Sweden and Finland of 350/100,000. In the bottom of each panel an estimate for the prevalence of snuff users is given as % of the population. The data for Sweden for time points 1988/9, 1996/7 and 2004/5, and the 2004/5 for Finland were obtained from [19]; all other estimates were from (www.pnlee.co.uk/ISS.htm)

Age-group specific analysis for male bladder cancer in Denmark, Sweden and Finland is shown in Fig. 2. The difference is seen in the two oldest age-group for which the increase towards 2016 is steep in Sweden while in Denmark and Finland a maximum in these age-groups is reached in the 1990s. The Danish rates in the oldest age-groups showed large rate fluctuations. In the bottom of the panels we show the estimates for the prevalence of snuff users (%) in the three countries.

In Table 1 we analyzed the estimated annual percent change (EAPC) in bladder cancer among Nordic men between 1995 (when the rates for Denmark and Finland peaked) and 2016. In age-groups 60 to 85+ years, the EAPCs for Denmark, Finland and Sweden were − 0.75, − 0.32 and 0.44%, respectively. The EAPC for Sweden was significantly different from that of Denmark and Finland (i.e., 95%CIs were non-overlapping). The increase in the EAPC for Sweden was steepest in the oldest age-group (1.42); this was significantly higher than the rates for Denmark and Finland.

**Table 1:** Case numbers and estimated annual percent change (EAPC) in incidence in age groups of male bladder cancer in Denmark, Finland and Sweden between 1995 and 2016

Country	Cases	EAPC [95%CI]
Age (years)		
**Denmark**		
60–85+	25,992	− 0.75 [− 1.03;-0.47]
60–69	8643	−1.11 [− 1.58;-0.64]
70–79	11,073	− 0.73 [− 1.07;-0.38]
80–85+	6276	0.16 [− 0.24;0.55]
**Finland**		
60–85+	14,208	− 0.32 [− 0.64;-0.01]
60–69	4157	− 0.17 [− 0.66; 0.34]
70–79	5914	−0.72 [− 1.28;-0.16]
80–89	4137	0.12 [− 0.32; 0.56]
**Sweden**		
60–85+	34,714	0.44 [0.22; 0.67]
60–69	9862	−0.07 [− 0.42; 0.28]
70–79	4494	0.57 [0.31; 0.82]
80–85+	10,358	1.42 [1.09; 1.74]

95%CI = 95% confidence interval

Some international studies compare bladder cancer rates between years 1990 and 2016, and we calculated EAPCs for these years (Table 2). The percent changes were for Denmark − 0.84 [− 1.03;-0.66], for Finland − 0.49 [− 0.71;-0.26] and for Sweden − 0.08 [− 0.25;0.08] [33]. The Swedish rate was close to 0 and not significant, in contrast to significantly declining rates for Denmark and Finland. These results calculated for period 1990 to 2016 from the EAPCs, were 14.3% for Denmark, 8.3% for Finland and 1.4% for Sweden (not significant).

**Table 2:** Case numbers and estimated annual percent change (EAPC) in incidence of male bladder cancer in the three Nordic countries between 1990 and 2016

Country	Cases	EAPC [95%CI]
Denmark	37,346	−0.84 [− 1.03;-0.66]
Finland	19,493	−0.49 [− 0.71;-0.26]
Sweden	47,534	−0.08 [− 0.25; 0.08]

95%CI = 95% confidence interval

Age-specific incidence data for bladder cancer are plotted at 10-year intervals (except for 2016) for Nordic males in Supplementary Fig. 1. The contrast in these graphs is largest for Finland (B) and Sweden (C); for Finland, the curves cluster together from year 1980 onwards, while for Sweden the rates increase in every 10-year period for age-groups 80 years and higher. The Danish rates for year 2000 and later showed no increase in the oldest age-groups.

## Discussion

The results showed that the incidence of bladder cancer has not decreased in Swedish men after 1990, as it has in Danish and Finnish men. This is in contrast to the Swedish male lung cancer rate which turned into decline after the early 1980s, and to the estimated population frequency of smoking men, declining from 30.3% (1980) to 11.1% (2006) [17]. This is also in contrast to the global trends of decline in male bladder cancer incidence, which between 1990 and 2016 were reported at 5.9% [33]. The decline has been 11.5% for highly developed countries, including Western Europe 13.9% and North America 6.5%. Our results for Denmark and Finland, calculated for the same period from the EAPCs, were 14.3% and 8.3%; the Swedish decline of 1.4% was not significant.

Why would the trend for the male Swedish bladder cancer differ from rates in the neighboring countries or from global development? A change in diagnostic or reporting practice may influence incidence rates, but no such change is known. A comparison to female rates is not helpful because Nordic women started to decrease their smoking habits later than men, and female lung and bladder cancer rates have been increasing until recently according to the NORDCAN data. Because of the low level of smoking among Swedish men, it is evident that the population attributable fraction of smoking for bladder cancer is declining, and other factors may exert a detectable role. Considering the lag time between smoking and bladder cancer of 20 to 40 years, smoking related bladder cancer should still be declining in Swedish men [3, 4]. Thus, new factor(s) appear to counteract this decline. We cannot find any more plausible explanations than the increasingly wide use of snuff by Swedish men.

Snuffing is a historical habit in Sweden but the habit decreased in popularity against cigarette smoking [34]. The amount of sold snuff reached the lowest level in the late 1960s but increased again and almost tripled by 2006. The habit was picked up fastest by young men and by 1988/89 29% of men aged 20 to 30 years were snuff users [19]. Swedish snuff is sold in two forms, poaches (small packages) and in loose form; these have been consumed in roughly equal proportions [34]. The average daily consumption in Sweden has been estimated at either 12 g of poached product or 30 g of loose product. Tobacco-specific nitrosamines (TSNAs), N-nitrosonornicotine (NNN) and 4-(methylnitrosamino)-1-(3-pyridyl)-1-butanone (NNK) have been measured in recent (since 2016) Swedish snuff products at the level of 0.5 microgram/g [35]. Thus, a daily consumer of loose snuff will ingest 15 micrograms/day after 2016, but ingested higher amounts earlier [35]. For comparison, TSNAs from a pack of low-tar cigarettes causes an exposure of 5 micrograms [36]. Thus, an average loose snuff user is currently exposed to 3 times higher levels to TSNAs than a pack-a-day smoker, and the difference was higher earlier [35]. TSNAs together with polycyclic aromatic hydrocarbons (constituents in tar) are assumed to be the most important carcinogens in tobacco smoke [37]. While the development of low-tar cigarettes reduced one carcinogenic component, it increased TSNA exposure [37].

Our age-group specific analysis showed that the increase in the Swedish bladder cancer incidence took place in the two oldest age groups, most steeply in the 80+ group (Fig. 2B). Those who were diagnosed in 2010 at the age of 80 were born in 1930. They belonged to the heavy smoking birth cohort, which however smoked less than their Danish and Finnish counterparts. Danes and Finns were able to turn down the increasing trend of bladder cancer for 70 to 79 year olds in contrast to Swedes whose incidence increased (Fig. 2A, C).

Based on published epidemiological studies, where snuff use has been assessed, the carcinogenic effects appear to be weak at best for the tumor types analyzed [20–23]. These studies have been conducted well and they include an individual assessment of snuff use, which however was usually available at the baseline only. The current snuffing habit is relatively new, after sales started to increase in the late 1960s (www.pnlee.co.uk/ISS.htm). According to sparse data from the latter source, consumers between 1970 and 1983 amounted to 13–16% of the male population. As the habit started among relatively young men (29% users among 20 to 29 year old in 1988/89) this cohort has reached age 40 to 49 by years 2008/09 [19]. It is true that even older men started with snuff but as the male median age for cancer in Sweden is over 70 years, it would be early to observe effects caused by an exposure confined to a fraction of the population [38]. While the generation of snuff users who never smoked is growing older, current epidemiological approaches may try and assess the possible interactions of dual use of cigarettes and snuff [39].

The limitation of the study is its ecological approach, as we have no information about individual snuff use. The implication is that we can only speculate about the causes of the observed trend changes. The strengths are the access to high quality national data sources and thus observation of trends in the entire population.

In conclusion, we observed an unexpected stabilization of Swedish male bladder cancer incidence after 1990, because it disagreed with the decreasing smoking prevalence and lung cancer rate among Swedish men. The stable bladder cancer trend was in contrast to declining trends in Denmark, Finland, Western Europe, North America and in the entire world. We suggest that this unique phenomenon may be related to increasing use of snuff by Swedish men. Average users of loose snuff ingest now 3 times more carcinogenic TSNAs than is inhaled by a smoker of one daily pack of cigarettes, and the difference was higher earlier before the reduction of the TSNA content in snuff. At the population level, snuff use in Sweden is relatively recent and the carcinogenic effects, if true, will become more evident in years to come.

## Supplementary Information

**Additional file 1 Supplementary Fig. 1**. Age-specific incidence data for bladder cancer plotted in 10-year intervals from 1960 onwards (except for 2016) for Danish (A), Finnish (B) and Swedish (C) males. The scale for y-axis for Sweden is different from the other countries.

## Data Availability

Publically available NORDCAN data can be accessed at (https://NORDCAN.iarc.fr/en/database#bloc2).
