# Supplementary material for: Incidence trends in bladder and lung cancers between Denmark, Finland and Sweden may implicate oral tobacco (snuff/snus) as a possible risk factor
Source: BMC Cancer. 2021 May 25;21:604. doi: 10.1186/s12885-021-08371-w (PMC8152093; doi:10.1186/s12885-021-08371-w)
Supplement: Supplementary file 1 — Additional file 1 Supplementary Fig. 1. Age-specific incidence data for bladder cancer plotted in 10-year intervals from 1960 onwards (except for 2016) for Danish (A), Finnish (B) and Swedish (C) males. The scale for y-axis for Sweden is different from the other countries. [file 12885_2021_8371_MOESM1_ESM.pptx]

## Slide 1
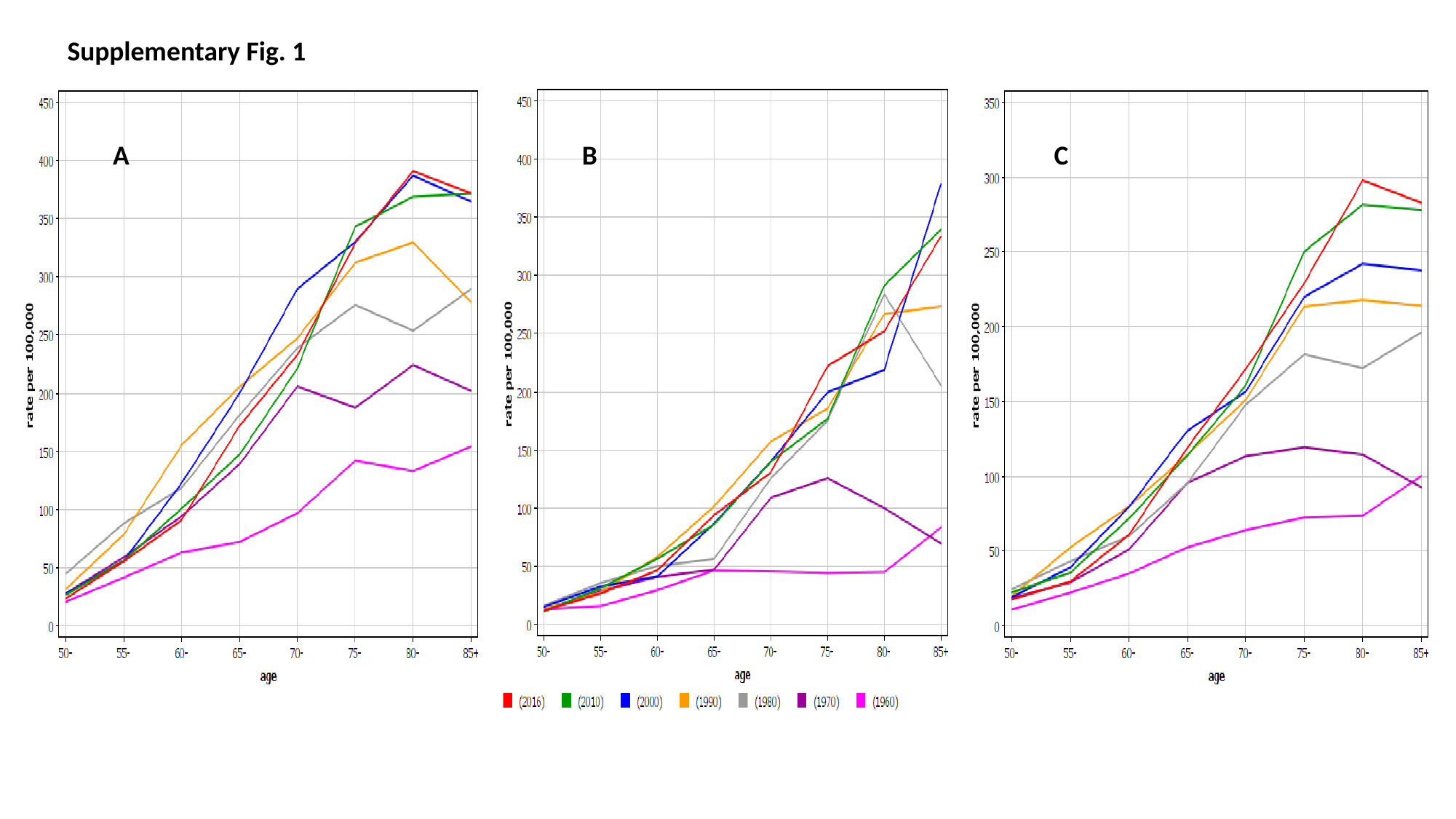

Supplementary Fig. 1
A
B
C

## Slide 2
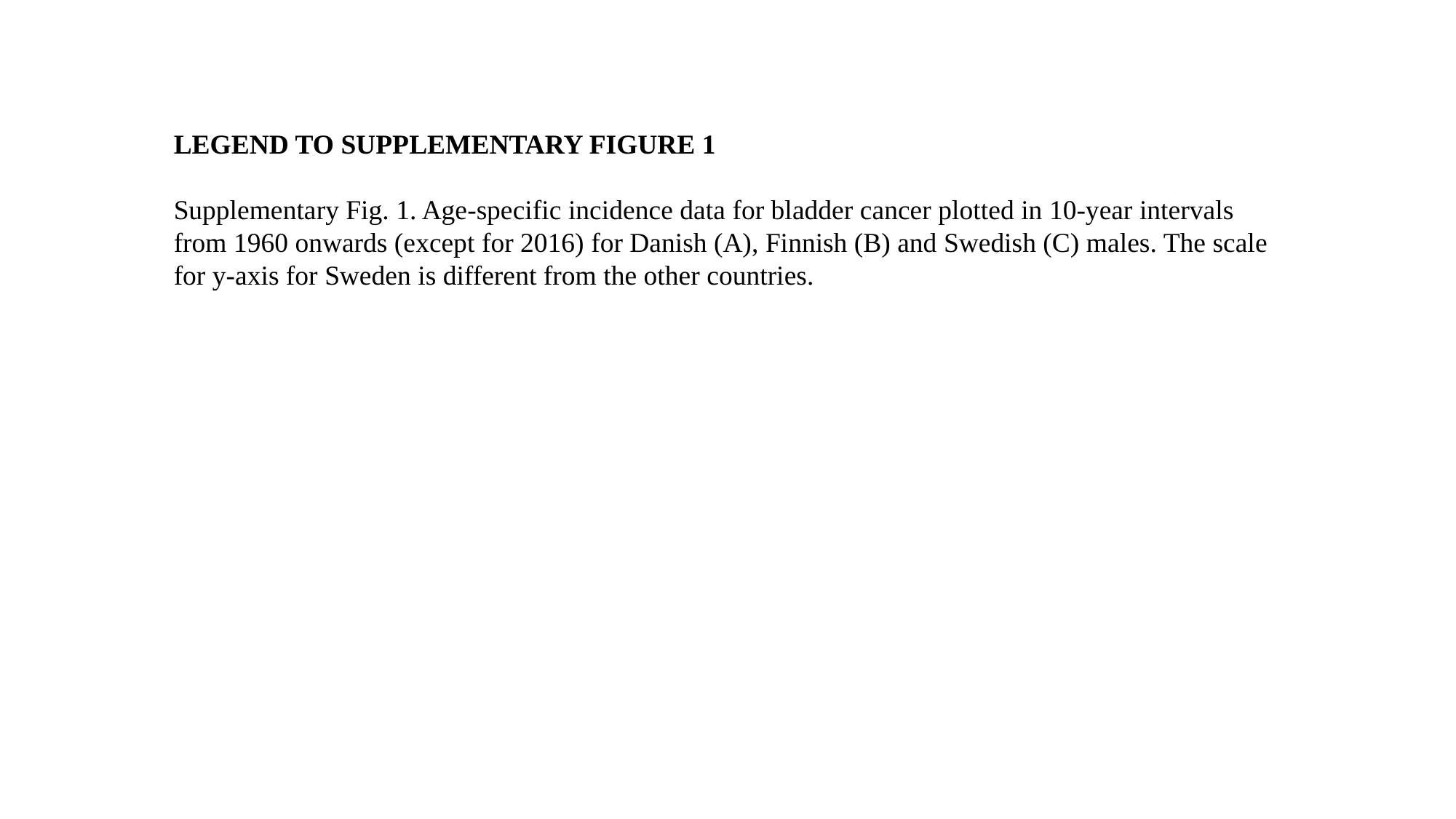

LEGEND TO SUPPLEMENTARY FIGURE 1
Supplementary Fig. 1. Age-specific incidence data for bladder cancer plotted in 10-year intervals from 1960 onwards (except for 2016) for Danish (A), Finnish (B) and Swedish (C) males. The scale for y-axis for Sweden is different from the other countries.
